# Divergent Serpentoviruses in Free-Ranging Invasive Pythons and Native Colubrids in Southern Florida, United States

**DOI:** 10.3390/v14122726

**Published:** 2022-12-06

**Authors:** Steven B. Tillis, Jillian M. Josimovich, Melissa A. Miller, Laura L. Hoon-Hanks, Arik M. Hartmann, Natalie M. Claunch, Marley E. Iredale, Tracey D. Logan, Amy A. Yackel Adams, Ian A. Bartoszek, John S. Humphrey, Bryan M. Kluever, Mark D. Stenglein, Robert N. Reed, Christina M. Romagosa, James F. X. Wellehan, Robert J. Ossiboff

**Affiliations:** 1 Department of Comparative, Diagnostic, and Population Medicine, College of Veterinary Medicine, University of Florida, Gainesville, FL 32608, USA; 2 U.S. Geological Survey, Fort Collins Science Center-South Florida Field Station in Everglades National Park, 40001 SR 9336, Homestead, FL 33034, USA; 3 Florida Fish and Wildlife Conservation Commission, Davie, FL 33314, USA; 4 Department of Microbiology, Immunology, and Pathology, College of Veterinary Medicine and Biomedical Sciences, Colorado State University, Fort Collins, CO 80521, USA; 5 Department of Biology, University of Florida, Gainesville, FL 32608, USA; 6 Department of Wildlife Ecology and Conservation, University of Florida, Gainesville, FL 32608, USA; 7 School of Natural Resources and Environment, University of Florida, Gainesville, FL 32608, USA; 8 U.S. Geological Survey, Fort Collins Science Center, Fort Collins, CO 80526, USA; 9 Conservancy of Southwest Florida, Environmental Science Department, Naples, FL 34102, USA; 10 U.S. Department of Agriculture, Animal and Plant Health Inspection Service, Wildlife Services, National Wildlife Research Center, Gainesville, FL 32641, USA

**Keywords:** Burmese python, colubrid, invasive, nidovirus, *Python bivittatus*, serpentovirus

## Abstract

Burmese python (*Python bivittatus*) is an invasive snake that has significantly affected ecosystems in southern Florida, United States. Aside from direct predation and competition, invasive species can also introduce nonnative pathogens that can adversely affect native species. The subfamily *Serpentovirinae* (order *Nidovirales*) is composed of positive-sense RNA viruses primarily found in reptiles. Some serpentoviruses, such as shingleback nidovirus, are associated with mortalities in wild populations, while others, including ball python nidovirus and green tree python nidovirus can be a major cause of disease and mortality in captive animals. To determine if serpentoviruses were present in invasive Burmese pythons in southern Florida, oral swabs were collected from both free-ranging and long-term captive snakes. Swabs were screened for the presence of serpentovirus by reverse transcription PCR and sequenced. A total serpentovirus prevalence of 27.8% was detected in 318 python samples. Of the initial swabs from 172 free-ranging pythons, 42 (24.4%) were positive for multiple divergent viral sequences comprising four clades across the sampling range. Both sex and snout-vent length were statistically significant factors in virus prevalence, with larger male snakes having the highest prevalence. Sampling location was statistically significant in circulating virus sequence. Mild clinical signs and lesions consistent with serpentovirus infection were observed in a subset of sampled pythons. Testing of native snakes (n = 219, 18 species) in part of the python range found no evidence of python virus spillover; however, five individual native snakes (2.3%) representing three species were PCR positive for unique, divergent serpentoviruses. Calculated pairwise uncorrected distance analysis indicated the newly discovered virus sequences likely represent three novel genera in the subfamily *Serpentovirinae.* This study is the first to characterize serpentovirus in wild free-ranging pythons or in any free-ranging North America reptile. Though the risk these viruses pose to the invasive and native species is unknown, the potential for spillover to native herpetofauna warrants further investigation.

## 1. Introduction

Burmese pythons (*Python bivittatus*) are native to southeast Asia but are an established invasive species in southern Florida, United States [1,2]. These pythons present a major predation pressure to native bird [3] and mammal species [4,5]. The pythons also compete with other predator species [5], applying further environmental pressures on native species. Besides direct competition and predation, pythons also represent a disease vector for native herpetofauna. Burmese pythons in their native range host a pentastome lung parasite (*Raillietiella orientalis*) which has since been documented in both the pythons and native snakes of Florida [6]. Some native species show greater susceptibility to the parasite than do Burmese pythons [7].

Florida has 44 native snake species including representation from colubrid, elapid, and viperid species [8,9]. In addition to environmental stressors such as habitat destruction [10,11], infectious diseases including ophidiomycosis (snake fungal disease) leave some native herpetofauna species at risk for population decline [12,13].

The viral order *Nidovirales* is a large and diverse assemblage of positive-sense, single stranded RNA viruses, with numerous significant human and animal pathogens including coronaviruses such as SARS-CoV-2 [14,15]. The subfamily *Serpentovirinae* (family *Tobaniviridae*) contains multiple viruses documented to infect reptiles. Serpentoviruses primarily cause disease of the oral cavity and respiratory tracts [16,17,18], but can infect a broader range of tissues [19]. The widest diversity of serpentoviruses has been documented in captive python species, but viruses have also been found in a number of boid, colubrid, lizard, and turtle species [18,20,21,22,23]. Serpentoviruses have also been associated with mortality events in wild populations of reptiles. In Australia, a novel serpentovirus in the endangered Bellinger River snapping turtle (*Myuchelys georgesi*) was identified in a mortality event of 400 turtles with respiratory disease [18]. A novel serpentovirus was also documented in wild shingleback skinks (*Tiliqua rugosa*) brought to wildlife rehabilitators for respiratory disease in Australia [22].

The aims of this project were to determine if serpentovirus was present in invasive Burmese pythons and native snakes in southern Florida, and if so to characterize the genetic diversity, clinical implications, and epidemiology of detected serpentoviruses.

## 2. Materials and Methods

### 2.1. Burmese Python Population and Sampling Investigation

To determine if serpentoviruses circulate in invasive Florida Burmese pythons, free ranging snakes were screened for the presence of serpentovirus. Oral swabs (n = 247) and tissue samples (n = 71) were collected from 246 Burmese pythons, all of wild southern Florida origin. Samples came from a variety of sources including long-term captive research animals and individuals sampled directly in the field. Pythons were sampled from across their invasive range in southern Florida during road surveys and opportunistic encounters. Samples were provided from various local, state, and federal organizations, including the United States Geological Survey (USGS), National Park Service (NPS), USDA, the Conservancy of Southwest Florida (CSF), and the Florida Fish and Wildlife Conservation Commission (FWC).

**Figure 1:**
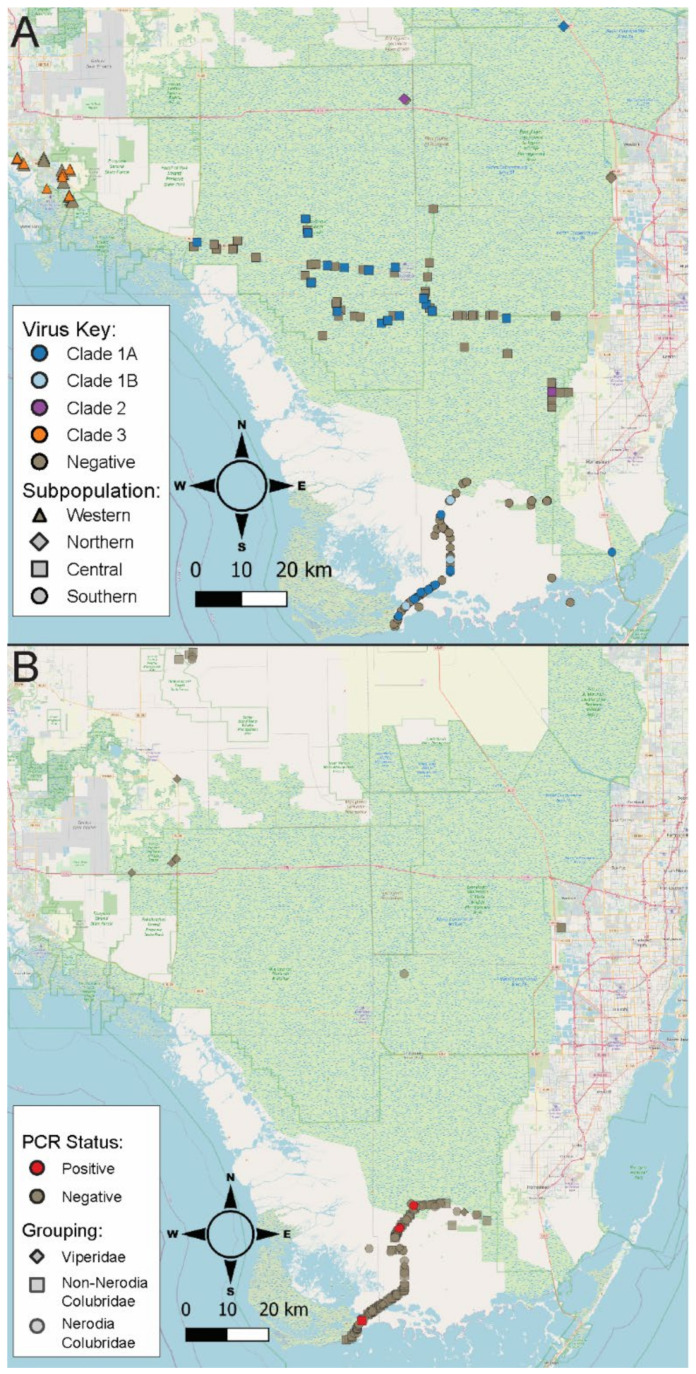
Maps of invasive Burmese python (**A**) and native snake (**B**) sampling and reverse transcription PCR (rtPCR) serpentovirus positive distribution in Florida, USA. Pythons of the western subpopulation had endemic virus clade 3, northern subpopulation clade 1A and clade 2, central subpopulation clade 1A and clade 2, and southern subpopulation clade 1A and endemic clade 1B.

When available, variables collected from submitted samples used for analysis included sampling date, sampling season (Summer/Fall/Winter/Spring), capture date, sample number (if tested more than once), reverse transcription PCR (rtPCR) result (positive/negative), virus type (categorical), sex (male/female), snout-vent length (cm), mass (g), oral mucosal appearance, capture coordinates (Universal Transversal Mercator [UTM] Northings [UTMx] and Eastings [UTMy]), and capture subpopulation designation (categorical). Only the initial samples from pythons with known or approximated capture coordinates sampled for virus within 14 days of capture were included in wild prevalence calculations. Wild pythons came from 4 major geographic clusters, considered hereafter as subpopulations (Figure 1A). Snakes from the western subpopulation include samples collected from areas around Naples, representing the western portions of the python range in the state. The southern subpopulation includes samples mainly collected in areas around Main Park Road in Everglades National Park, as well as other samples from the southern extreme of the python range in Everglades National Park. The central subpopulation includes samples from Big Cypress National Preserve, northern portions of Everglades National Park, and other wilderness areas bordering the Tamiami Trail. Finally, the northern subpopulation represents samples collected from the northern extreme of the Burmese python range in the state in areas around Everglades and Francis S. Taylor Wildlife Management Area. Sample distribution maps were created in Q GIS v3.2.22 (https://qgis.org) (accessed on 2 January 2021).

All statistical calculations for the study were performed in Rstudio v2021.09.2 (https://rstudio.com) with package vcd V1.4–10. Association between rtPCR result and sex, and rtPCR result and season were determined with a Chi-squared analysis with an alpha of 0.05. Association between rtPCR result and mass, rtPCR result and snout-vent length, and rtPCR result and latitude was determined using a logistic regression model with an alpha of 0.05. Finally, the association between virus clade/subpopulation and rtPCR result/subpopulation was determined with a Fisher’s Exact Test in 4 × 4 and 2 × 4 contingency tables, respectively, and a significance of 0.05.

### 2.2. Longitudinal Sampling

A subset of 44 sampled pythons were retained after capture and tested for serpentovirus by rtPCR two or more times for a total of 116 longitudinal samples. There was no standard sampling interval between tests.

### 2.3. Native Snake Population and Sampling Investigation

In total, 219 swabs from a total of 18 native snake species were screened for serpentovirus (Table 1). Two hundred-one samples from 17 native snake species were collected during road surveys within the southern portion of Everglades National Park (Figure 1B) as part of a joint project by USGS, NPS and University of Florida (UF). Other native snake samples included 18 samples from 7 snake species, collected using road surveys or radio telemetry by FWC. Sampling by FWC was scattered across areas of the greater southern Florida region south of Lake Okeechobee. Sample distribution maps were created in Q GIS v3.2.22 (https://qgis.org) (accessed on 2 January 2021).

Variables collected from submitted native snake samples included species (categorical), rtPCR result (positive/negative), capture coordinates (UTMx and UTMy) and general notes. Associations between the rtPCR result and notes of oral reddening were determined with a Fisher’s Exact Test with an alpha of 0.05.

### 2.4. Postmortem Examinations

Complete postmortem examinations were performed on seven serpentovirus rtPCR positive Burmese pythons. All snakes were euthanized using AVMA approved euthanasia protocols [24]. Samples of all tissues were collected and preserved in 10% neutral buffered formalin. Samples of the liver, kidney, lung, spleen, trachea, esophagus, feces, and an oral swab were collected and archived frozen (−80 °C). After a minimum of 24 h fixation, tissues were processed routinely, cut at 5 µm sections, and stained with hematoxylin and eosin (H&E).

### 2.5. Serpentovirus rtPCR Screening

Samples tested by rtPCR included both oral tissue and oral swabs. Oral tissue samples were collected from 71 Burmese pythons euthanized by captive bolt [24]. Euthanized snakes were decapitated; heads were stored frozen (−20 °C), and later thawed overnight prior to tissue extraction. Tracheal, esophageal, and tongue tissue were pooled from each animal in 1 mL of TRIzol (Thermo Fisher, Ambion Life Technologies, Carlsbad, CA, USA). Tissues were extracted using RNA clean and concentrator columns (Zymo Research, Irvine, CA, USA) using protocols described in Hoon-Hanks et al., 2018 [16]. All remaining samples consisted of oral swabs (either Rayon or cotton-tipped plastic shaft), collected by running the swab along the oral mucosa at the labial margin, the trachea, the choana, and the caudal oral cavity and cranial esophagus. Swabs were placed in sterile microcentrifuge tubes or 15 mL conical tubes containing RNA*later* Stabilization Solution (Invitrogen, Carlsbad, CA, USA) and kept on ice until frozen (−20 °C). A cold chain was maintained in transporting the swabs to the University of Florida. Upon receipt, samples were either stored at −80 °C until RNA extraction or were extracted immediately using the Zymo Quick-RNA MiniPrep Kit (Zymo Research, Irvine, CA, USA), per manufacturer’s recommendation.

For initial screening, samples were subjected to a modified rtPCR protocol described in Hoon-Hanks et al., 2019 [20] using sense primer BarniPVTF and antisense primer BarniDYTR. To produce longer amplicons for phylogenetic analysis, additional Burmese python serpentovirus-specific primers were designed (Table 2). The rtPCR mix for each primer pair included 4 µL of 10 µM forward/sense primer, 4 µL of 10 µM reverse/antisense primer, 25 µL of 2x PCRBio rt-PCR mix, 11.5 µL of H_2_O, 2.5 µL of 20Xrtase Taq, and 3 µL RNA extract. Samples were run in a MJ Research PTC-100 Thermal Cycler with conditions for each as follows: 50 °C for 10 min; 94 °C for 2 min; 94 °C for 30 s, primer pair specific annealing temperature for 30 s, and 72 °C for 30 s for 40 cycles; and 72 °C for 7 min followed by holding at 4 °C.

Products of rtPCR were run on a 1% agarose gel. Bands of target length were excised and nucleic acids were extracted using Zymo Clean Gel DNA Recovery Kit (Zymo Research, Irvine, CA, USA) per the manufacturer’s recommendation. Samples were submitted for bidirectional Sanger sequencing to a commercial facility (Genewiz, South Plainfield, NJ, USA). Sequences were edited and aligned using Geneious Prime (Auckland, New Zealand) and considered positive if a serpentovirus sequence was returned as the closest match on NCBI BLASTN.

### 2.6. Illumina MiSeq Nextgen Sequencing

To capture genome-wide variations of genetic sequence in novel serpentoviruses, a subset of representative samples, including nine positive python samples and three positive native snake samples, was subjected to Illumina MiSeq next-generation sequencing. Previously extracted RNA was concentrated using Zymo Clean RNA Clean and Concentrator kit (Zymo Research, Irvine, CA, USA). Samples were prepped by depleting ribosomal RNA using NEBNext rRNA Depletion Kit (Human/Mouse/Rat) (NEBNext, Ipswich, MA, USA) according to manufacturer’s recommendations supplemented with AMPure XP beads (NEBNext, Ipswich, MA, USA). Libraries were generated using NEBNext Ultra II RNA Library Prep kit (NEBNext, Ipswich, MA, USA) using the manufacturer’s protocols. Finally, prepped samples were pooled and loaded into an Illumina 600 cycle V3 MiSeq cartridge (Illumina Inc., San Diego, CA, USA) and run on an Illumina MiSeq system. Contigs of generated reads were assembled De Novo in CLC Genomics Benchtop software (Version 20, CLC BIO). Sequences generated from the project were deposited in the GenBank database and sequence read archive (SRA). Sequences generated from Sanger and Illumina MiSeq sequencing can be found in Genbank accession numbers [MZ971274–MZ971355, ON256215–ON256216] and data from Illumina MiSeq sequencing in BioProject accession number PRJNA753790.

### 2.7. Phylogenetic Analysis and Preliminary Taxonomic Classification

**Figure 2:**
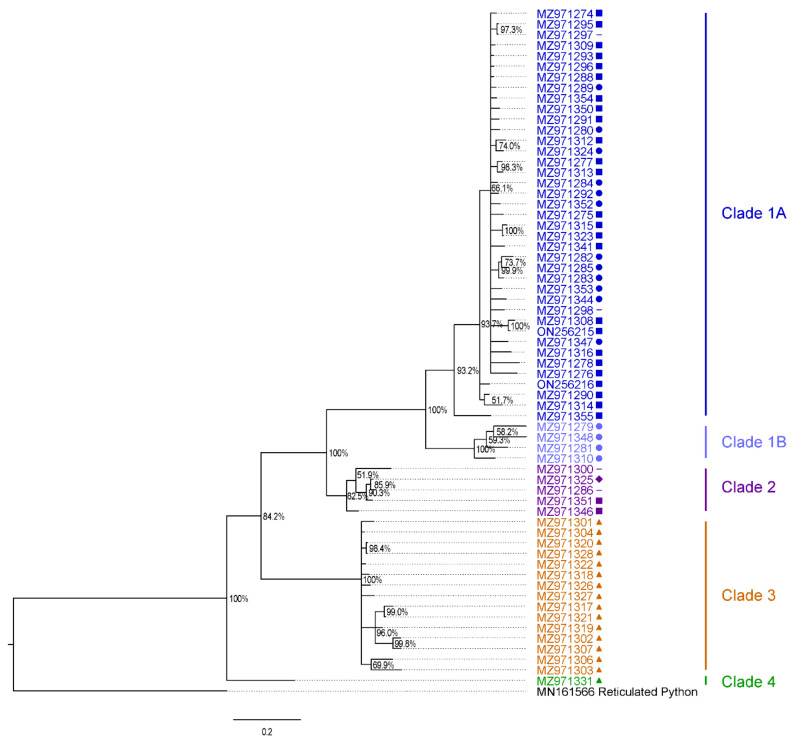
Bayesian phylogenetic tree of a 369-nucleotide fragment of the ORF1b gene from novel Burmese python serpentoviruses from southern Florida, USA. The Burmese python serpentoviruses formed 5 clades: clade 1A (navy blue), clade 1B (light blue), clade 2 (purple), clade 3 (orange), and clade 4 (green). Samples came from 4 subpopulations within the state of Florida, USA: western (triangle), northern (diamond), central (square), and southern (circle). A previously described reticulated python serpentovirus (MN161566) was used as an outgroup. Bayesian posterior probabilities are shown at branch points. The scale at the bottom represents expected substitutions per site.

Sequences generated using Sanger sequencing and next-generation sequencing were subjected to phylogenetic analysis. To address diversity within Burmese python serpentoviruses, a 369-nucleotide (nt) region of the ORF1b gene coding for the RNA-dependent RNA polymerase, and shared between the amplicons of different primer sets was targeted. Nucleotide sequences from novel Burmese python serpentoviruses were aligned along with a closely related reticulated python (*Python reticulatus*) serpentovirus as an outgroup (Genbank MN161566) using multiple alignment using fast Fourier transform (MAFFT) [25]. Genbank accession numbers are referenced in Figure 2.

**Figure 3:**
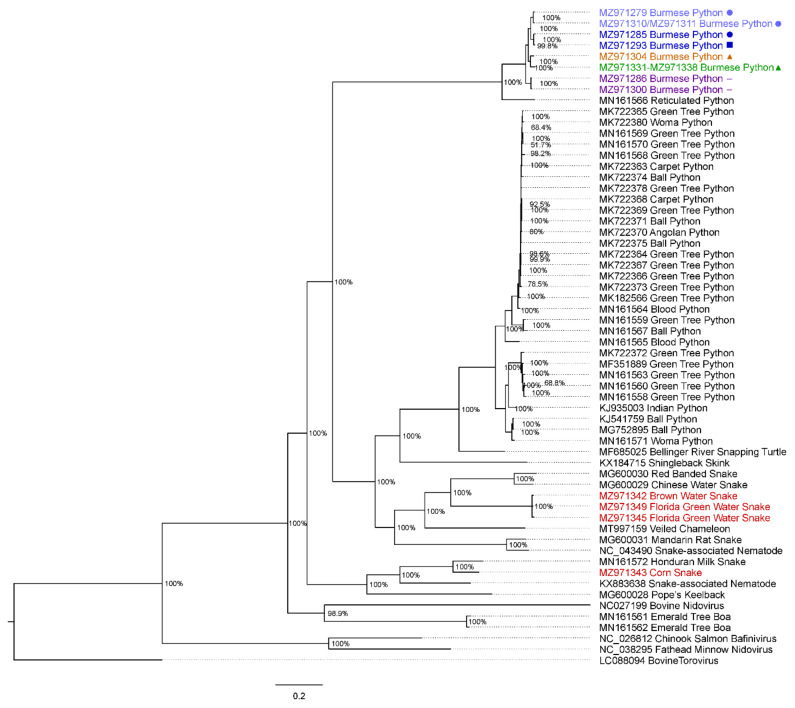
Bayesian phylogenetic tree of the translated ORF1b gene of southern Florida, USA snake serpentoviruses. Novel Burmese python serpentoviruses are highlighted as follows: clade 1A (navy blue), clade 1B (light blue), clade 2 (purple), clade 3 (orange), and clade 4 (green). Samples came from 4 subpopulations within the state of Florida, USA: western (triangle), northern (diamond), central (square), and southern (circle). Highlighted in red are novel Florida native colubrid serpentoviruses. In black text are previously described viruses published to GenBank. Bovine Torovirus (LC088094) was used as an outgroup. Bayesian posterior probabilities are shown at branch points. The scale at the bottom represents expected substitutions per site.

To examine the relatedness of discovered Burmese python and native snake viruses to other serpentoviruses, Illumina MiSeq reads of the ORF1b gene from a subset of samples were translated and compared to a wider group of 44 published serpentovirus genomes as well as related bovine nidovirus, fathead minnow nidovirus, chinook salmon bafinivirus, and bovine torovirus as an outgroup. The entire translated ORF1b gene was aligned separately using MAFFT. For select viruses without full coverage of the ORF1b gene, gaps were inserted between contigs as aligned to reference genome reticulated python serpentovirus. Genbank accession numbers are referenced in Figure 3.

For both nucleotide and amino acid analyses, a Bayesian method of phylogenetic inference (Mr. Bayes 3.2.7a with gamma distributed rate variation, 4 chains of 2.5 × 10^6^ generations with 25% burn-in) was performed on the CIPRES server [26,27,28]. Phylogenetic trees were visualized using FigTree software (http://tree.bio.ed.ac.uk/software/figtree/) (accessed on 8 January 2021).

As a preliminary assessment of how the detected virus sequences could be classified, we calculated pairwise uncorrected distances (PUD) between the new sequences and the most closely related existing sequences. PUD values were determined from the distance matrix of alignments of pp1ab sequences from the pp1a/b junction to the end of the DEAD-like helicase C domain (corresponding to residues 5797–7177 in YP_009052475).

### 2.8. Virus Isolation Attempts

To attempt to isolate novel Burmese python serpentoviruses, three tracheal swab samples and three lung tissue samples from five unique snakes were inoculated onto established reptile cell culture lines. Diamond python heart, Burmese python heart, and amethystine python splenic fibroblast cells, established from *Morelia spilota, Python bivittatus,* and *Simalia amethistina* respectively, were used for inoculations for all samples. All cell lines were maintained in 32 °C incubators in a humidified, 5% CO_2_ atmosphere. Cells were grown in T25 flasks using Minimum Essential Medium with Earle’s Balanced Salts, L-Glutamine (MEM/EBSS; GenClone), 10% Heat Inactivated fetal bovine serum (FBS; GenClone), nonessential Amino Acids (Caisson), penicillin-streptomycin solution (GenClone), amphotericin B (HyClone), and gentamicin (GenClone). Cell monolayers were grown until 90–95% confluency was reached, at which time media was removed, and the flask was washed twice using sterile phosphate buffered saline (PBS) prior to inoculation.

For tissue inoculations, lung tissue was finely minced using scalpel blades and mixed with 1200–2400 μL of completed medium. For swab inoculations, 1200–2400 μL of completed medium was added along with the swab to a 15 mL centrifuge tube and vortexed for 10 s. For mock inoculations, untreated completed media was used. A flask of each cell line was inoculated with 400 μL of tissue, swab, or mock treated media. Flasks were incubated for 60 min at room temperature with gentle rocking every 10 min. Complete culture medium (4 mL) was added to each flask after the initial adsorption period, and returned to a 32 °C, humidified 5% CO_2_ atmosphere. Flasks were observed for cytopathic effect every other day. For P1 inoculations, 400 μL of P0 cell lysate was inoculated onto fresh flasks of the same cell line and the procedure detailed above was repeated. Lysate from P1 flasks was screened for serpentovirus using the rtPCR protocol previously described.

## 3. Results

### 3.1. Burmese Python Population and Sampling Investigation

A total of 318 Burmese python samples, representing all pythons sampled, were screened for serpentoviruses by rtPCR, and 27.8% (88/318) of samples tested positive. Of the 246 unique snakes tested, 31.3% (77/246) of pythons had at least one positive test. Samples from 172 individual pythons were considered for wild prevalence calculations. Results from these samples are shown in Table 3. The rtPCR serpentovirus prevalence in wild pythons was 24.4% (42/172).

In free-ranging pythons, viral prevalence was higher in male than female snakes, with 34.3% (35/102) of males testing positive compared to 12.1% (7/58) of females (Chi-Square; *p* = 3.9 × 10^−3^, n = 160). Higher viral prevalence was also found in larger pythons. The average snout-vent length (SVL) of positive snakes (n = 42) was 234.0 ± 17.5 cm 95% confidence interval (CI), compared to 184.7 ± 14.7 cm in negative pythons (n = 130; (logistic regression; *p* = 1.2 × 10^−3^). Likewise, there was a similar trend in snake mass, with a heavier average mass of a positive snake (9955.8 g ± 3324.3 95% CI) in comparison to negative snakes compared to (7091.6 g ± 1952.7) although not statistically significant (*p* = 1.6 × 10^−1^, n = 172). When examined independently via logistic regression, longer (*p* = 8.2 × 10^−4^, n = 102) and heavier (*p* = 6.1 × 10^−3^, n = 102) male pythons were positive. Longer female pythons were positive (*p* = 4.2 × 10^−2^, n = 58) but there was no relationship between virus prevalence and female mass (*p* = 5.3 × 10^−2^, n = 58).

In free-ranging pythons, lower viral prevalence was observed in the warmer spring and summer seasons, although those differences were not statistically significant (Chi-Square; *p* = 7.3 × 10^−2^, n = 172). Lowest viral prevalence was documented in summer (17.4%; n = 15/86), followed by spring (24.4%; 10/41), fall (36.4%; 4/11), and winter (38.2%; 13/34). Snakes sampled in the summer and fall seasons had a smaller average SVL length, which coincides with the summer hatching season for pythons [29]. Snakes sampled in summer had an average SVL of 153.4 cm (n = 86), compared to 204.8 cm (n = 11) in fall, 231.0 cm (n = 34) in winter, and 256.8 cm (n = 41) in the spring.

Positive Burmese python samples from both free-ranging and captive snakes contained a diverse assemblage of novel, divergent serpentoviruses that were most closely related to a reticulated python serpentovirus (Genbank MN161566). Across 64 unique Burmese python viral sequences, viruses shared 75.6% to 81.3% nucleotide identity with the reticulated python serpentovirus across a common 369 nt region of ORF1b gene. Burmese python viruses shared 81.3–100% pairwise identity in this region. Results from a Bayesian phylogenetic analysis of the 369-nucleotide fragment of the Burmese python serpentovirus are shown in Figure 2.

Of the nine python samples subjected to MiSeq sequencing, amplification of large portions of the genome was successful in eight samples. A summary of nucleotide fragments generated by MiSeq sequencing are shown in Supplementary Table S1. All sequenced viruses produced portions or complete coverage of the ORF1ab gene. The spike (S) gene, ORF3 Putative transmembrane protein, matrix (M) gene, nucleocapsid (N) gene, and ORF6 hypothetical protein was generated in six of eight sequenced Burmese python viruses (Supplementary Table S1).

Bayesian phylogenetic analysis of the full-length, translated ORF1b gene sequence also showed the amplified Burmese python serpentoviruses formed a clade with the captive reticulated python serpentovirus (Genbank MN161566; Figure 3) in the genus *Septovirus* with a Bayesian posterior probability of 100%. However, the Burmese python viruses were distant and basal to members of the serpentovirus genus *Pregotovirus* that contains viruses found in other captive pythons such as green tree python nidoviruses, first described in *Morelia viridis,* or ball python nidovirus, with a Bayesian posterior probability of 100% (Figure 3).

Burmese python serpentoviruses formed five unique clades that we classified as 1A, 1B, 2, 3, and 4 (Figure 2 and Figure 3). These viruses were not distributed equally in sampled subpopulations (Figure 1A). Among wild positive snakes, 69.0% (29/42) were infected with clade 1A viruses, 7.1% (3/42) clade 1B, 4.8% (2/42) clade 2, and 19.0% (8/42) clade 3. Because clade 4 was only detected on subsequent sampling and not an initial swab, it is not represented in the wild viral prevalence. All of the Burmese python virus sequences were separated by <4% PUD, consistent with them being classified into one species [30]. These sequences are separated by ~19% PUD from the most closely related existing sequences, from reticulated pythons (MN161566). This PUD falls between the previously established cutoffs for subgenus (13–14%) and genus (35–36%), indicating that the identified Burmese python clades could constitute a new genus in the subfamily *Serpentovirinae*.

Both prevalence of virus and distribution of virus type (Figure 1A) varied across python subpopulations. Western subpopulation snakes had a viral prevalence of 34.8% (8/23), with all positive samples containing clade 3 viruses, the only clade detected in this subpopulation (Figure 2). The northern subpopulation had a viral prevalence of 40.0% (2/5), with viruses from clade 1A and clade 2 each being detected in a single snake, respectively (Figure 2). However, viral prevalence for the northern subpopulation should be interpreted with caution due to the very low sample size from this region (n = 5). The central subpopulation had a total prevalence of 22.1% (17/77), with 20.8% (16/77) of snakes testing positive for clade 1A, and 1.3% (1/77) of snakes testing positive for clade 2 (Figure 2). The southern subpopulation had a prevalence of 22.4% (15/67), with 17.9% (12/67) of samples testing positive for clade 1A, and 4.5% (3/67) testing positive for clade 1B. Clade 1B was only represented by animals from the southern subpopulation (Figure 1A). The distribution of virus clades varied across subpopulations (Fisher’s Exact Test; *p* = 6.1e-09, n = 172), but there was not a difference in total overall virus prevalence between subpopulations (Fisher’s Exact Test, *p* = 4.3 × 10^−1^, n = 172; Table 3).

### 3.2. Clinical Signs of Infection

**Figure 4:**
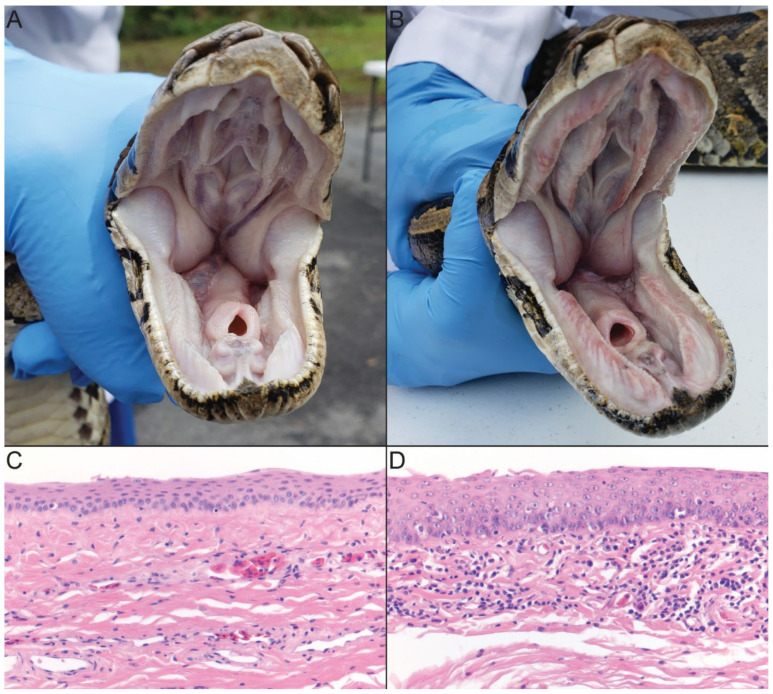
Gross and histologic appearance of the oral cavity of serpentovirus reverse transcription PCR (rtPCR) negative (**A**,**C**) and positive (**B**,**D**) Burmese pythons (*Python bivittatus*) from southern Florida, USA. In contrast to the gross appearance of a serpentovirus rtPCR negative Burmese python (**A**), snakes rtPCR positive for serpentovirus (**B**) variably showed reddening of the oral mucosa, particularly along the margins of the teeth, as well as increased amounts of mucoid oral material. Histologically, in comparison to the oral mucosa of a rtPCR negative Burmese python (**C**), snakes rtPCR positive for serpentovirus showed thickening of the oral mucosa (hyperplasia) as well as increased numbers of submucosal and mucosal lymphocytic infiltrates (**D**). Photomicrographs of H&E-stained oral mucosa at 400× total magnification.

Serpentovirus positive pythons did not show consistent gross signs of viral infection. Some positive pythons presented with slightly thickened oral mucus secretions and reddened oral mucosa (Figure 4B). However, these signs were highly variable between positive and negative snakes and did not reliably correlate with infection status. None of the pythons that tested positive for novel Burmese python serpentoviruses showed clinical signs of respiratory disease.

### 3.3. Postmortem Findings

On gross examination, significant lesions were restricted to the oral cavity and included reddening of the oral mucosa and increased amounts of tacky mucoid secretions in 4/7 rtPCR positive snakes. Microscopically, the most significant finding was the presence of mild lymphocytic inflammation and mucosal hyperplasia within the oral cavity of 6/7 snakes (Figure 4D). Mild lymphocytic inflammation was also observed in the proximal esophagus (4/7), nasal mucosa (2/7), and tongue (1/7). Other findings included coelomic granulomas (4/7), as well as intestinal (2/7) and respiratory (1/7) endoparasitism. Necropsied snakes were rtPCR positive for either clade 1A or 2 viruses.

### 3.4. Longitudinal Sampling

Longitudinal analysis was performed on 116 oral swabs from 44 pythons originally removed from the wild but retained across multiple research colonies and sampled two or more times. Results for these 44 snakes are shown in Table 4. Although there was no standard testing interval, intervals between samples ranged from 4 days to 193 days, with an average of 43 days between tests.

Individual snakes frequently converted between testing positive and negative. At least one rtPCR positive sampling occurred in 52.3% (23/44) of longitudinally sampled snakes. Of those, 73.9% (17/23) also had at least one negative test. A total of 10 snakes had multiple positive tests, with eight of those snakes maintaining 100% viral nucleotide identity between positive tests. Snake 28 is of interest as it initially tested positive but then had three negative tests in the next 4 months before again testing positive (Table 4), maintaining 100% viral nucleotide identity across a 133 nt fragment. Exceptions to viral identity include snake 12 which had two single nucleotide substitutions across a 411 nt region which did not change amino acid sequence, and snake 32 which retained only 96.7% identity across a 196 nt region. Also of note are snakes 38, 39, 40 and 41, which went from testing negative to testing positive within the same month. Sequence identity between these four samples ranged from 97.8% to 86.4% across a 436 nt region.

### 3.5. Native Snake Sampling

Given the relatively high serpentovirus prevalence within free-ranging Burmese pythons, there was concern about potential spillover of viruses into native reptiles. A total of 219 samples from 18 native snake species representing 13 genera were screened for serpentovirus (Table 1). The majority of these samples came from Everglades National Park (Figure 1B).

While no native reptile samples tested positive for any Burmese python serpentoviruses, five snakes were positive for other novel, divergent serpentoviruses. Four of the positive samples were found in two species of watersnake, the brown watersnake (*Nerodia taxispilota*) and the green watersnake (*N. floridana*) (Table 1). All positive *Nerodia* were found within 6.6 km of each other, with three positive samples coming from within only 350 m of each other. Furthermore, out of the 219 native snake samples, nine snakes (all *Nerodia*) were noted as having some degree of oral discoloration. Of the nine *Nerodia* observed with oral discoloration, 22.2% (2/9) tested positive for a novel serpentovirus, compared to only 2.2% (2/93) of *Nerodia* with no noted oral discoloration; this association was statistically significant (Fisher’s Exact Test, *p* = 4.0 × 10^−2^, n = 101). The fifth positive native snake was a corn snake (*Pantherophis guttatus*) with a virus divergent from both python and *Nerodia* serpentoviruses; this snake was found 24 km south of the positive *Nerodia* (Figure 1B). Overall viral prevalence in native species was too low to determine how it varies between species and genera, as positive samples were only detected in two *Nerodia* and one *Pantherophis* species (Table 1).

Large genomic portions of all four native snake viruses were sequenced. Nucleotide sequence for the S gene, ORF3 Putative transmembrane protein, M gene, N gene, and ORF6 hypothetical protein was generated in all three water snake viruses (Supplementary Table S1). For the corn snake virus, ORF1ab and S gene sequences were generated.

Bayesian phylogenetic analysis of the entire ORF1b gene revealed that native snake serpentoviruses fell outside both the common captive snake serpentoviruses in the genus *Pregotovirus* and the Burmese python serpentovirus clades (Figure 3). The *Nerodia* virus clade fit among other colubrid serpentoviruses such as red banded snake serpentovirus (Genbank MG600030) and Chinese water snake serpentovirus (Genbank MG600029) in the genus *Lyctovirus* along with veiled chameleon serpentovirus (Genbank MT997159) in the genus *Vebetovirus*, all with Bayesian posterior probabilities of 100% (Figure 3). The three *Nerodia* virus sequences were separated by <2% PUD from each other and by ~37% PUD from the most closely related database sequences (MG600029-30). This indicates that these viruses may constitute a new genus and possibly a higher order taxon. In comparison, the corn snake serpentovirus appears in an entirely separate clade from the *Nerodia* viruses, matching more closely with viruses in the genus *Infratovirus*, including Honduran milk snake virus (Genbank MN161572) and Xinzhou Toro-like virus (Genbank KX883638; Figure 3). Bayesian posterior probabilities were also 100%. The corn snake virus sequence was separated by 17% PUD from the Honduran milk snake virus, the nearest database sequence, consistent with establishment of a new genus.

### 3.6. Virus Isolation

Unfortunately, despite using both swab and tissue samples from five unique snakes representing multiple virus clades (1A, 2 and 3) on multiple unique reptile cell lines, virus isolation attempts were unsuccessful.

## 4. Discussion

This study documents the presence of divergent serpentoviruses in free-ranging, invasive Burmese pythons (*P. bivittatus*) and native colubrids in southern Florida. Burmese python serpentoviruses were found in 24.4% (42/172) of tested pythons and fell into five major clades that were unevenly distributed across the invasive range of the python. In the southern subpopulation, virus clades 1A and closely related 1B were the only viruses detected, and clade 1B was not found in other regions sampled. While virus clade 2 had the lowest detected prevalence, it was found across a wide area of the state including northern and central subpopulations. In the western subpopulation, virus clade 3 was the sole virus clade detected in wild python samples, and this clade was not detected elsewhere. Interestingly, pythons from the western subpopulation also exhibit genetic and phenotypic characteristics absent in other pythons throughout the invasive range, perhaps indicative of a unique introduction event [31,32].

A potential limitation of comparisons between viral prevalence among differing clades of serpentovirus is the unknown and potentially variable sensitivity and specificity of the utilized rtPCR. Although the Burmese python serpentoviruses group together phylogenetically, significant differences in nucleotide sequence made rtPCR amplification of larger viral sequences challenging, even with specific primers based on data from MiSeq genome sequencing. Therefore, it is possible that screening of samples with the primers used in this study may have missed divergent viruses. Moreover, there were likely different primer binding efficiencies between viral clades that may have affected rtPCR results. It is also possible that variations in sampling technique or storage and shipping conditions from the different submitting organizations could have affected the detected prevalence.

The wild rtPCR prevalence of 24.4% (42/172) and total study prevalence of 27.8% (88/318) in southern Florida Burmese pythons are similar to prevalence data seen in captive python populations. A serpentovirus prevalence of 37.7% (156/414) has been observed in captive pythons in the United States [20], and a prevalence of 30.7% (438/1426) has been observed in captive pythons in Europe [23]. Despite a similar overall viral prevalence to those reported in captive snakes, the novel viruses found in the Burmese pythons are highly divergent from commonly seen captive python viruses. Moreover, the reported prevalence in the studies in captive snakes is likely skewed by testing of animals that showed clinical signs of suspect viral disease. Together, the differences in viral sequence and clinical condition limit direct comparisons between captive and wild python viral prevalence.

Serpentovirus rtPCR positive pythons in this study tended to be larger (234.0 cm) than negative pythons (184.7 cm). One interpretation of this finding is that older, larger pythons have more exposure time than smaller, younger pythons and may be more likely to contract serpentoviruses, a trend also observed in captive pythons by Hoon-Hanks et al., 2019 [20]. Viral prevalence in male Burmese pythons was also nearly three times higher than females in the study. This may either indicate a difference in susceptibility or a difference in viral transmission rates between the sexes. Burmese pythons in Florida are known to form breeding aggregations during the breeding season, with documentation of up to eight males attempting to copulate with a single female python [33]. Such breeding aggregations might result in higher rates of intrasexual male-to-male viral transmission than intersexual transmission. Additionally, male Burmese pythons are also known to have larger home ranges than females [34], which could increase the number of exposure opportunities with other pythons.

There was also a variation in prevalence across seasons, with the lowest prevalence detected during warmer periods of the year such as summer and fall. While changes in detected prevalence could be affected both by a seasonal influx of neonate pythons and behavior changes of adult animals in breeding season, other factors could play a compounding role in viral transmission. During cooler months, immunosuppressive stressors on ectothermic subtropical pythons could lead to increased viral susceptibility or replication. Conversely, during warmer periods of the year longevity of an RNA virus in the environment could be limited by higher temperatures and humidity [35].

In longitudinally sampled Burmese pythons, detection of virus by rtPCR was inconsistent. While over half of the longitudinally sampled pythons tested positive at least once, the overall testing prevalence was similar to the viral prevalence among free-ranging pythons. This indicates either reinfection events or variable levels of viral shedding below the threshold of a positive rtPCR result. Although the significance of identity between sequences of snakes with multiple positive tests is limited by short amplicon sizes, the observed nucleotide identity in eight of 10 snakes supports variable rates of shedding rather than complete clearance of infection during negative tests. Further research is needed to determine if complete clearance of serpentovirus is possible, but captive python data likewise indicated a possibility of intermittent viral shedding [20].

In vitro isolation attempts for the Burmese python serpentoviruses were unsuccessful, despite using multiple snake cell lines, including a Burmese python heart cell line, and using samples representing multiple virus clades (Clades 1A, 2 and 3). In contrast, in vitro experimental inoculations of ball python nidovirus show cytopathic effect when inoculated on python cell lines [16]. While further research and successful isolation would be necessary to confirm, this finding may represent limited host species range or cell type fidelity in Burmese python serpentoviruses.

No wild pythons in the study displayed clinical signs of lower respiratory infection or died of respiratory disease within the capture period. Either during sample collection or postmortem examination, a subset of rtPCR positive pythons exhibited only reddened oral mucosa with increased mucoid secretions. Histologic lesions in necropsied snakes were mild in nature and limited primarily to lymphocytic inflammation and mucosal proliferation in the oral cavity, with lesser involvement of the esophagus and nasal cavity. Though the lesions in the examined Burmese pythons were mild, they were consistent with those previously reported in captive snakes with serpentovirus infection [16,23,36,37,38]. Moreover, other studies of serpentoviruses in captive snakes have documented a high asymptomatic viral prevalence with minimal clinical signs of infection [20,23]. In further support of this, a subset of python rtPCR results from this study included in the analysis of Claunch et al., 2022 indicated no association between metrics of python stress and rtPCR result [39].

While serpentovirus-positive free-ranging pythons in this study appeared to suffer little clinical consequence from their infection, the risk the viruses may pose to native herpetofauna is unclear. Burmese python serpentoviruses were not found during sampling of native herpetofauna, which may be due to limitations of the study. Some native species were underrepresented or absent in our sample pool, which likely greatly limited viral detection of viruses potentially circulating at a low prevalence in native snakes. Additionally, most sampling was conducted in the Everglades National Park region, which represents only a portion (southern subpopulation: virus clade 1A and 1B) of the viral genetic diversity seen across the python range in the state. However, to date, serpentovirus transmission between snake families has not been documented in captive snakes [20,23].

The discovery of additional divergent serpentoviruses in snakes native to Florida was surprising. The overall viral prevalence in native snakes (2.3%, 5/219) was much lower than that observed in Burmese pythons (24.4%, 42/172), and only five individuals representing two genera (*Nerodia* and *Pantherophis*) were rtPCR positive. The two clades of viruses from native snakes were highly divergent from both Burmese python and captive python viruses, supporting entirely separate viral origins between the pythons and native snakes. Though the clinical significance of the novel identified viruses is unclear, two of the positive water snakes were noted as having oral discoloration or scabbing.

The diverse assemblage of ophidian serpentoviruses discovered in this study are divergent from previously described serpentoviruses. The Burmese python serpentoviruses form a highly divergent and previously underrepresented group of python serpentoviruses. While these viruses group together with a reticulated python serpentovirus in the genus *Septovirus*, PUD analysis indicates these viruses may warrant the creation of a new genus in the subfamily *Serpentovirinae,* However, while five clades of Burmese python serpentoviruses were identified in this study, the low PUD (<4%) separating these viruses is most constituent with their classification as a single species. The two clades of colubrid virus described also likely constitute novel species, but that are different enough from other published serpentoviruses that they will likely necessitate the creation of new genera. The *Nerodia* serpentoviruses formed a genus-like clade related to viruses in the genus *Lyctovirus* documented in wild Chinese colubrid species and more basal veiled chameleon serpentovirus in the genus *Vebetovirus*. Interestingly, veiled chameleons (*Chamaeleo calyptratus*) are an established exotic reptile species in southern Florida [40]. Given the high PUD (~37%) that separated this clade from the most closely related database sequences, it is likely these viruses constitute a new genus if not a higher order taxon. Meanwhile, the corn snake serpentovirus was placed into a comparatively basal clade of colubrid viruses. While some of the most closely related viruses are members of the genus *Infratovirus,* the results of the PUD analysis were consistent with the corn snake serpentovirus likely representing a novel *Serpentovirinae* genus. 

This study identifies and describes five clades of novel Burmese python serpentovirus and two novel clades of colubrid serpentoviruses from free-ranging snakes in Florida. This represents the first characterization of serpentoviruses in free-ranging pythons globally and free-ranging reptiles in North America. Additional research may improve prediction of potential clinical consequences of any given serpentovirus infection, but this study demonstrates that serpentoviruses can maintain host–pathogen equilibrium at high viral prevalence in wild snake populations without clear evidence of clinical disease.

## Figures and Tables

**Table 1:** Florida native snake species sampled and tested by reverse transcription PCR (rtPCR) for serpentovirus. The ‘Sampled’ column represents the number of samples and proportion to total samples for each genus and species (same row if single species is represented). Likewise, the ‘rtPCR Positive’ column represent the number and serpentovirus prevalence via rtPCR for each genus and species.

Genus	Species	Common Name	Sampled	rtPCR Positive
*Agkistrodon*	*A. conanti*	Florida Cottonmouth	5 (2.3%)	-
*Cemophora*	*C. coccinea*	Scarlet Snake	20 (9.1%)	-
*Coluber*	*C. constrictor*	Southern Black Racer	4 (1.8%)	-
*Crotalus*	*C. adamanteus*	Eastern Diamondback Rattlesnake	6 (2.7%)	-
*Diadophis*	*D. punctatus*	Ringneck Snake	8 (3.6%)	-
*Drymarchon*	*D. couperi*	Eastern Indigo	1 (0.5%)	-
*Lampropeltis*			6 (2.7%)	-
	*L. getula*	Eastern Kingsnake	4 (1.8%)	-
	*L. elapsoides*	Scarlet Kingsnake	2 (0.9%)	-
*Nerodia*			102 (46.6%)	4 (3.9%)
	*N. fasciata*	Banded Watersnake	31 (14.2%)	-
	*N. taxispilota*	Brown Watersnake	27 (12.3%)	2 (7.4%)
	*N. floridana*	Florida Green Watersnake	18 (8.2%)	2 (11.1%)
	*N. clarkii*	Salt Marsh Snake	26 (11.9%)	-
*Pantherophis*			29 (13.2%)	1 (3.4%)
	*P. guttatus*	Corn Snake	21 (9.6%)	1 (4.8%)
	*P. alleghaniensis*	Eastern Ratsnake	8 (3.7%)	-
*Liodytes*	*L. alleni*	Striped Crayfish Snake	9 (4.1%)	-
*Storeria*	*S. dekayi*	DeKay’s Brown Snake	3 (1.4%)	-
*Thamnophis*			26 (11.9%)	-
	*T. sirtalis*	Garter Snake	11 (5.0%)	-
	*T. saurita*	Ribbon Snake	15 (6.8%)	-
Total	18		219	5 (2.3%)

**Table 2:** Primers used for reverse transcription PCR (rtPCR) targeting novel Burmese python serpentoviruses in Florida, USA with annealing temperature and target amplicon size.

Sense Primer	Antisense Primer	Annealing Temp (°C)	Target Length (Approx. nt)
BarniPVTF (5′-GAG GAC TCC ACA ARC CAG TCA C-3′)	BarniDYTR (5′-RCT RCG GTC GCA TTT CGT RTA RTC-3′)	46	150
BurmLHHF1 (5′-TCG AGG ACT TCA AAG CCG TC-3′)	BurmLHHR1 (5′-TGT TCG TCG TTG GGT GTT GA-3′)	42	650
BurmROF1 (5′-CTC ATG TCM GTC AAR CAA GAC GAC AT-3′)	BurmROR2 (5′-AAR CAA AAD GCW GCC ATC TC-3′)	42	450
BurmSTF1 (5′-CAA GGY CTC ATG TCA GTC AA-3′)	BurmSTR3 (5′-AAR GCW GTN GTY GCR TCY CCT GA-3′)	46	550

**Table 3:** Epidemiological data from 172 wild southern Florida, USA Burmese pythons sampled for serpentovirus via reverse transcription PCR (rtPCR). For continuous variables, 95% confidence intervals (95% CI) are included.

Variable	Category	Positive	Negative	*p* Value	df	Cramér’s V
Wild Prevalence	(95% CI)	42 (24.4%) ± 6.5%	130 (75.6%) ± 6.5%	-	-	-
Subpopulation ^1^	Western	8 (34.8%)	15 (65.2%)	4.3 × 10^−1^	3	0.117
	Northern	2 (40.0%)	3 (60.0%)			
	Central	17 (22.1%)	60 (77.9%)			
	Southern	15 (22.4%)	52 (77.6%)			
Sex ^2^	Male	35 (34.3%)	67 (65.7%)	3.9 × 10^−3^ *	1	0.243
	Female	7 (12.1%)	51 (87.9%)			
Season ^2^	Winter	13 (38.2%)	21 (61.8%)	7.3 × 10^−2^	3	0.196
	Spring	10 (24.4%)	31 (75.6%)			
	Summer	15 (17.4%)	71 (82.6%)			
	Fall	4 (36.4%)	7 (63.6%)			
Morphometrics ^3^	Mass (g) (95% CI)	9955.8 ± 3324.3	7091.6 ± 1952.7	1.6 × 10^−1^	171	0.968
	SVL (cm) (95% CI)	234.0 ± 17.5	184.7 ± 14.7	1.2 × 10^−3^ *	171	0.943
Latitude ^3^	Northing (95% CI)	2,839,487 ± 12133	2,832,197 ± 5758	2.4 × 10^−1^	171	0.981

* Statistically Significant, ^1^ Fisher’s Exact Test, ^2^ Chi-Square Test, ^3^ Logistic regression.

**Table 4:** Longitudinal serpentovirus reverse transcription PCR (rtPCR) results of 44 Burmese pythons tested two or more times originating from central, southern, and western subpopulations in southern Florida, USA. Positive tests are denoted by a green “+”, and negative tests an orange “−”. No snakes were tested in the months of May and June 2019. Viral clade (Virus) is shown for snakes with positive tests. In snakes with multiple positive diagnostic tests, the shared fragment length of positive diagnostic Sanger sequencing and corresponding percent of nucleotide identity are also shown.

SubPop	Virus	Snake	Oct	Nov	Dec	Jan		Feb		Mar		Apr	Jul	Aug	Sep	nt Identity between + Tests	Fragment Length (nt)
**Central**	1A	1								−	+						
		2	+					−		+						100%	133
		3			+			−									
		4			−	+				−							
		5												+	−		
		6			+	+		−	−							100%	133
		7						+		−							
		8								+		+				100%	133
		9			+	+										100%	133
		10						+				+				100%	133
		11				+		−									
		12				+				+	−					99.5%	411
	2	13											+		−		
	NA	14	−			−				−		−					
		15						−		−	−						
		16				−				−	−		−				
		17				−				−							
		18				−		−									
		19				−		−									
		20		−		−		−		−							
		21						−		−							
		22				−		−									
		23				−		−		−	−		−				
		24						−	−				−				
		25						−	−				−				
		26				−		−		−		−					
		27						−		−							
Southern	1A	28		+		−	−	−		+			−			100%	133
		29		+		+		+								100%	133
		30						−					+				
		31												+	+	100%	133
		32				+		+								96.7%	196
	1B	33				−		+									
	NA	34			−	−	−	−		−							
		35		−				−									
		36						−					−				
		37												−	−		
Western	3	38								−	+						
		39								−	+						
		40								−	+						
	4	41								−	+						
	NA	42								−	−						
		43								−	−						
		44								−	−						

## Data Availability

Sequences generated from Sanger and Illumina MiSeq sequencing can be found in Genbank accession numbers [MZ971274–MZ971355, ON256215–ON256216] and data from Illumina MiSeq sequencing in BioProject accession number PRJNA753790. Snake epidemiological data are available in Tillis et al., 2022 [41].

## Supplementary Materials

The following supporting information can be downloaded at: https://www.mdpi.com/article/10.3390/v14122726/s1, Table S1: MiSeq Sequencing Results.
