# Supplementary material for: Divergent Serpentoviruses in Free-Ranging Invasive Pythons and Native Colubrids in Southern Florida, United States
Source: Viruses. 2022 Dec 6;14(12):2726. doi: 10.3390/v14122726 (PMC9782103; doi:10.3390/v14122726)
Supplement: Supplementary file 1 [file viruses-14-02726-s001.zip › viruses-1974441_Supplementary_Table S1.pdf]

viruses

MDPI

Supplimentary Table S1. Summary of MiSeq next generation sequence in novel Burmese python and Florida snake viruses. Raw generated reads can be found in BioProject accession number PRJNA753790, with specific BioID's listed below alongside associated Genbank accession numbers from sequnced fragments, fragment length, and location of the fragment in the genome.

Virus Name	BioID	Genbank	nt length	Genome Location
FL Green Watersnake Serpentovirus sp. R191890	SAMN20708797	MZ971349	30,317	ORF1ab, S gene, ORF3 Putative transmembrane protein, M gene, N gene, ORF6 hypothetical protein
Burmese Python Serpentovirus sp. R19815	SAMN20708798	MZ971330	726	ORF1ab
		MZ971331	4,590	ORF1ab
		MZ971332	5,533	ORF1ab
		MZ971333	1,672	ORF1ab
		MZ971334	742	ORF1ab
		MZ971335	3,123	ORF1ab
		MZ971336	2,775	ORF1ab
		MZ971337	616	ORF1ab
		MZ971338	1,748	ORF1ab
		MZ971339	750	ORF1ab
		MZ971340	831	ORF1ab
Burmese Python Serpentovirus sp. R19232	SAMN20708799	MZ971304	14,184	ORF1ab
		MZ971305	6,453	ORF1ab
Burmese Python Serpentovirus sp. R19223	SAMN20708800	MZ971299	4,404	ORF1ab
		MZ971300	15,315	ORF1ab, S gene, ORF3 Putative transmembrane protein, M gene, N gene, ORF6 hypothetical protein
Burmese Python Serpentovirus sp. R19199	SAMN20708801	MZ971293	18,557	ORF1ab
		MZ971294	6,519	ORF1ab, S gene, ORF3 Putative transmembrane protein, M gene, N gene, ORF6 hypothetical protein
Burmese Python Serpentovirus sp. R191808	SAMN20708802	MZ971286	20,205	ORF1ab, S gene, ORF3 Putative transmembrane protein, M Gene, N gene, ORF6 hypothetical Protein
		MZ971287	6,475	ORF1ab
Burmese Python Serpentovirus sp. R19424	SAMN20708803	MZ971310	8,896	ORF1ab
		MZ971311	6,738	ORF1ab, S Gene, ORF3 Putative transmembrane protein, M gene, N gene, Orf6 hypothetical protein

Cornsnake Serpentovirus sp. R191271	SAMN20708804	MZ971343	26,412	ORF1ab, S gene
FL Green Watersnake Serpentovirus sp. R191278	SAMN20708805	MZ971345	30,320	ORF1ab, S Gene, ORF3 Putative transmembrane protein, M gene, N gene, Orf6 hypothetical protein
Brown Watersnake Serpentovirus sp. R191234	SAMN20708806	MZ971342	27,011	ORF1ab, S Gene, ORF3 Putative transmembrane protein, M gene, N gene, Orf6 hypothetical protein
Burmese Python Serpentovirus sp. FNP18		MZ971279	23,491	ORF1ab, S gene, ORF3 Putative transmembrane protein, M Gene, N gene, ORF6 hypothetical Protein
Burmese Python Serpentovirus sp. FNP59		MZ971285	20,328	ORF1ab, S gene, ORF3 Putative transmembrane protein, M Gene, N gene, ORF6 hypothetical Protein
